# Do it right the first time; implementation of three-dimensional technology for bone defect reconstruction in upper extremity surgery

**DOI:** 10.1016/j.jham.2025.100390

**Published:** 2025-11-25

**Authors:** Hedayatullah Esmati, Tim de Jong, Kim Y. Jochem, Vincent M.A. Stirler, Rob F.M. van Doremalen, Hinne A. Rakhorst

**Affiliations:** a Department of Plastic, Reconstructive and Hand Surgery, Medical Spectrum Twente, Enschede, the Netherlands; b Department of Plastic, Reconstructive and Hand Surgery, Radboud University Medical Center, Nijmegen, the Netherlands; c Radboudumc 3D Lab, Radboud University Medical Center, Nijmegen, the Netherlands; d Department of Trauma Surgery, Radboudumc, Nijmegen, the Netherlands; e Military Health Organisation, Ministry of Defence, Kromhout Kazerne, Utrecht, the Netherlands; f Medical 3D Lab, Department of Medical Technology, Medical Spectrum Twente, Enschede, the Netherlands; g Department of Plastic, Reconstructive and Hand Surgery, University Medical Center Groningen, Groningen, the Netherlands

**Keywords:** 3D technology, Patient-specific, Upper extremity, Virtual planning

## Abstract

Three-dimensional (3D) technology has rapidly evolved from an innovative concept into an essential surgical tool. The increasing availability of high-resolution imaging, affordable 3D printers and user-friendly software has accelerated the integration of 3D technology into clinical practice, including upper extremity surgery. Despite the growing interest, many surgeons remain uncertain about how to practically implement this technique in clinical workflows. This review illustrates the clinical use of 3D technology in upper extremity bone defect reconstruction. We highlight both the preoperative and intraoperative applications of 3D technology, illustrated by detailed case examples. We also provide a practical guideline to support clinicians in adopting 3D technology as part of their routine surgical practice.

## Abbreviations:

AI: Artificial Intelligence
CT: Computed tomography
CTA: Computed tomography angiography
MC: Metacarpal
MC1: First metacarpal
MT2: Second metatarsal
PACS: Picture archiving and communication system
VSP: Virtual surgical plan
XR: Extended Reality
3D: Three-dimensional

## 1 Introduction

As surgeons, our aim in surgery is to be specific, reproducible and efficient, while minimizing complications. In order to achieve these aims, the ‘do it right the first time’ strategy from lean manufacturing principles can be extrapolated to surgical practice. An important advancement in the treatment of complex three-dimensional (3D) bone defects is the use of 3D techniques, including virtual surgical planning and the creation of patient-specific guides.

Three-dimensional technology has rapidly evolved from an innovative concept into an essential surgical workflow. Its initial medical application began in the field of craniofacial surgery by Mankovich et al. who demonstrated the clinical utility of 3D printing in 1990 by creating skull models from computed tomography (CT) scans.^1^ This breakthrough significantly enhanced surgeons’ ability to visualize and prepare complex craniofacial reconstructions. By the mid-1990s, 3D computer models and 3D printed prototypes were used to plan bone graft and prosthetic reconstructions.^2^ These early successes in craniofacial surgery paved the way for adaptation in trauma and orthopedic surgery, with the first orthopedic applications emerging around the year 2000. Since then, 3D printing has been increasingly integrated into orthopedic surgery, especially for complex trauma and reconstructive procedures.^3^

The widespread availability of high-resolution CT scans has significantly advanced the integration of 3D technologies into clinical practice, including in upper extremity surgery. Additionally, the increasing affordability of 3D printers and the development of user-friendly software, combined with the growing availability of in-hospital medical 3D labs with specialized teams, have enabled more hospitals to routinely adopt these advanced technologies. Despite increasing interest and promising outcomes, many surgeons remain uncertain about the practical steps involved in implementing 3D technology into their clinical workflows. This paper describes how to implement the clinical use of 3D technology in upper extremity bone defect reconstruction through detailed case examples. Both preoperative and intraoperative applications are specifically highlighted. Furthermore, practical guidelines are provided to assist clinicians in integrating 3D technology into their daily practice.

## 2 Preoperative applications of 3D technology

The use of 3D technology in the preoperative phase allows surgeons to accurately assess complex pathologies and precisely plan surgical procedures.^4^ This application is particularly valuable in cases of complex bone deformities, malunions and extensive bone defects, where 3D imaging may aid in capturing and conveying spatial complexity better than conventional CT scans. High-quality imaging of the patient's anatomy is the cornerstone of any 3D reconstruction. Typically, thin-slice CT scans (with a slice thickness of ≤1.0 mm) are obtained for both the affected and contralateral uninjured limbs. These scans are then converted into digital 3D reconstructions using specialized software to precisely show the detailed bone structure. The contralateral side serves as a template for reconstruction. Using a mirroring technique, where the scan of the contralateral limb is digitally mirrored to create a symmetrical model, the surgical team (often in collaboration with biomedical engineers or technical physicians) can virtually reconstruct the missing segments with accuracy. When a CT scan of the contralateral side is unavailable, a virtual reconstruction of the missing bone fragments can be estimated either from averaged anatomical data from comparable patient populations or based on the experience of the available expert (e.g., surgeon or senior technical physician).

Once accurate 3D models are available, virtual surgical planning enables the simulation of the intended surgical outcome. This includes fracture reduction, osteotomy planning, correction of malunited segments, and positioning of osteosynthetic material. Additionally, it allows for a detailed assessment of bone defects, estimation of the extent of bone debridement and preoperative optimization of implant positioning. This virtual rehearsal helps surgical teams evaluate the feasibility of planned interventions and anticipate potential intraoperative challenges. In reconstructive procedures involving bone transplantation, detailed imaging of donor sites (e.g., iliac crest, fibula, scapula) is performed to generate virtual 3D models for graft harvest. These models are digitally adjusted to precisely fit the defect, minimizing the need for intraoperative adjustments, enhancing reconstruction accuracy and minimizing donor site morbidity. For instance, in forearm reconstruction using vascularized fibula grafts, virtual modeling of the fibula can improve anatomical alignment and surgical efficiency, thereby reducing the operation time.^5^

Furthermore, virtual surgical planning can be complemented by producing life-size 3D-printed anatomical models. These tangible models are particularly useful for preoperative selection and precise contouring of osteosynthesis plates, ensuring better anatomical fit and reducing intraoperative modifications. This approach also reduces the intraoperative use of fluoroscopy.^6^

## 3 Intraoperative applications of 3D technology

The intraoperative use of 3D technology appears to enhance surgical precision and reduce variability in upper extremity reconstructions. Patient-specific products derived from preoperative virtual surgical planning include patient-specific guides (such as drilling, osteotomy and reduction templates), pre-selected and pre-contoured osteosynthesis plates, and patient-specific implants. These techniques can assist in the accurate execution of complex surgeries. Additionally, 3D-printed anatomical models may serve as intraoperative references, aiding spatial orientation during complex reconstructions involving multi-fragmentary fractures or severe deformities.

Patient-specific guides conform precisely to the patient's anatomy. In the upper extremity, including the scaphoid, distal radius, ulna or humerus, these guides facilitate accurate bone osteotomies, drill trajectories, bone reductions, and implant placement through predefined surgical angles and depths for optimal anatomical alignment and bone healing outcomes.^7^ For example, patient-specific drill guides enhance the accuracy of fracture fixation by aligning drills along predefined paths, optimizing screw insertion and angulation.

Pre-contoured osteosynthesis plates and patient-specific implants further optimize intraoperative precision and efficiency. In standard practice, osteosynthesis plates often require manual contouring during surgery, which can be a time-consuming and technically demanding process, especially in peri-articular fractures or osteotomies. Using patient-specific 3D-printed bone models as templates, plates can now be accurately pre-bent prior to surgery.^8^

Another important yet often underappreciated benefit of preoperative 3D planning is its positive impact on intraoperative workflow and surgeon working conditions. By making critical decisions regarding the surgical approach and instrument selection preoperatively, procedures become more structured, predictable, and streamlined. This not only reduces stress during critical phases of the surgery but also enhances the overall efficiency and focus of the surgical team.

## 4 Challenges

Although 3D technology offers many benefits for both preoperative planning and intraoperative procedures, several limitations must be acknowledged. As previously discussed, 3D-printed anatomical models usually rely on bone-specific CT imaging and, therefore, do not provide sufficient information about the surrounding soft tissues, blood vessels and nerves. This could be a limitation, particularly in surgeries where accurate identification and preservation of neurovascular structures are critical. However, in selected cases, additional CT angiography (CTA) can be performed to visualize and segment relevant vascular structures as part of the 3D model. Moreover, despite utilizing high-resolution thin-slice CT scans, the accuracy of 3D reconstructions can be compromised by metal artifacts, osteoporotic bone, or residual deformities from previous fractures. These factors can obscure anatomical details and reduce the precision of segmentation and modeling. Furthermore, the time required to generate 3D models poses a considerable challenge in acute surgical care settings. In an ideal setting, the process from CT image acquisition to a finalized 3D model takes approximately 4–6 h during working hours, making it impractical for emergency procedures, where rapid clinical decision-making is essential. Lastly, effective clinical integration of 3D technology requires specific training and experience. Surgeons and operative teams must become proficient in the basic principles of this technique, learn to handle patient-specific guides, and incorporate digital workflows into their surgical routines. The associated learning curve can slow down broader adoption, particularly in low-volume or resource-limited settings. However, adopting a technical physician or biomedical engineer into the surgical team can greatly accelerate implementation, streamline the workflow, and reduce the learning curve for the surgical team.

## 5 Guideline for centers implementing 3D technologies in surgical practice

The integration of 3D technologies in surgical care transforms preoperative planning, allowing for more precise and individualized interventions. To facilitate successful implementation of 3D technologies, hospitals must consider a variety of strategic, technical and regulatory factors. This includes deciding whether to establish an in-house medical 3D lab, mainly focused on imaging, segmentation, and virtual surgical planning, or outsourcing everything to external providers. While an in-house facility offers faster workflows, enhanced interdisciplinary collaboration, and long-term cost efficiency, external providers can supply certified, regulation-compliant devices such as patient-specific guides and implants. For most hospitals, a hybrid approach may be the most efficient, as it offers an ideal balance between flexibility and legal and safety compliance. In-hospital medical device production is subject to regulatory frameworks. These frameworks may allow hospitals to produce certain medical devices in-house under specific conditions that can vary across regions. Usually, hospitals are required to ensure device safety and performance through proper documentation and quality management before in-house medical device production is permitted. Under such regulatory frameworks, anatomical 3D models, both virtual and physical, can often be developed in-house, as well as 3D virtual surgical planning. In many cases, patient-specific guides can be designed in-house. However, due to regulatory and quality requirements, such as being able to sterilize the guide and material compliance, outsourcing the 3D printing of patient-specific guides to a certified medical provider is often more efficient. In contrast, patient-specific implants are generally subject to stricter regulations and are therefore more often fully designed and produced by externally certified medical providers who comply with relevant market authorizations. Regulations and practical approaches for implementing 3D technologies in healthcare may vary between countries. Therefore, hospitals should tailor their strategies to comply with the applicable local laws and standards.

Implementation of 3D technologies requires significant technological infrastructure, specialized segmentation and 3D planning software, and 3D printers suited to the desired clinical applications. In addition, access to imaging data and secure storage of all working files are crucial. Beyond technology, the success of 3D technology integration depends heavily on the collaborative involvement of surgeons, technical physicians or clinically oriented biomedical engineers and 3D printing experts. Each of these experts plays a key role in the workflow of 3D applications in surgical care. Surgeons define the clinical objectives. Then, together with the technical physician, the suitability of 3D technologies for the intended treatment is assessed and the 3D anatomical models are evaluated for accuracy. Subsequently, the surgeon approves the virtual surgical plans and designs of the patient-specific guides. Technical physicians, or other professionals with comparable expertise, are responsible for developing 3D anatomical models, performing virtual surgical planning, and designing patient-specific guides. Close collaboration and frequent communication between surgeons and technical physicians are essential to ensure that the applied 3D technology accurately reflects the clinical needs and treatment goals. Lastly, 3D printing experts manage the printing process that converts virtual components of the 3D technologies, such as anatomical models, patient-specific guides or implants, into physical products that meet the requirements for quality and clinical usability.

**Fig. 1:**
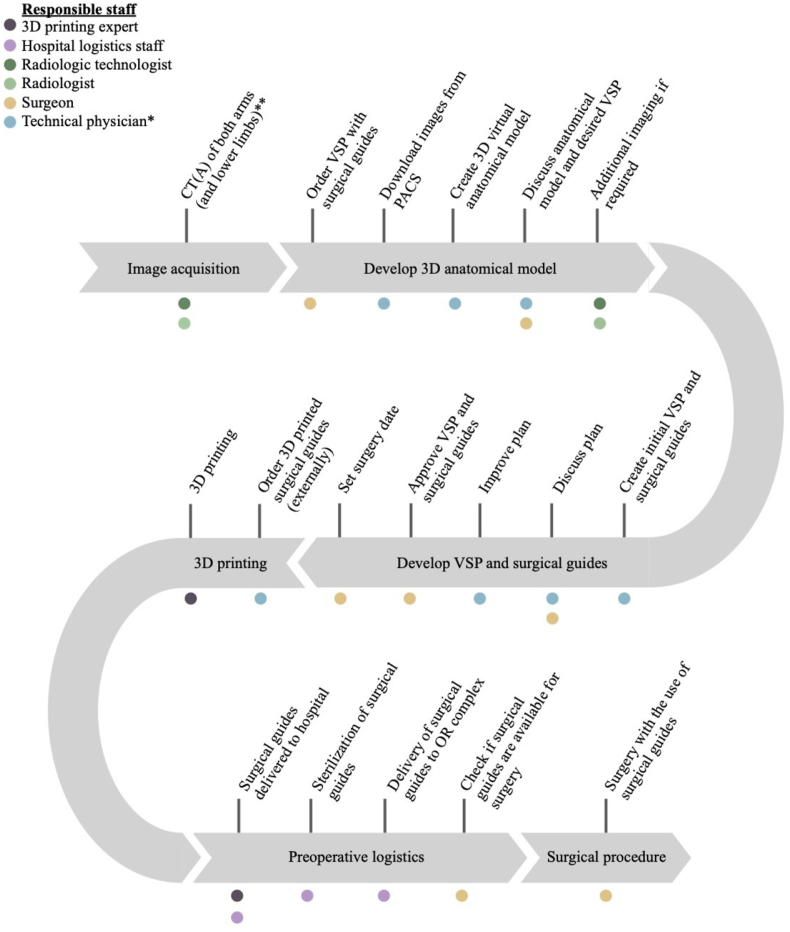
**Workflow** Example of a streamlined workflow for implementing virtual surgical planning and surgical guides in upper extremity reconstruction surgery. A hybrid approach is applied in this workflow, combining in-house 3D lab expertise with outsourced manufacturing. Colored circles denote the roles and responsibilities of the various experts involved at each step of the workflow. Abbreviations: CTA – computed tomography angiography; OR – operating room; PACS – picture archiving and communication system; VSP – virtual surgical plan. **∗**Technical physician or equivalent expert, such as a clinically oriented biomedical engineer. **∗∗** CTA when angiography is indicated; CT(A) of lower limbs if fibula transfer is considered; CT(A) requirements: slice thickness <1.0 mm, no soft filter, metal artifact reduction applied if prior osteosynthesis material is present in situ.

While having the right technological infrastructure and collaborative involvement of experts are essential for the integration of 3D technology in surgical care, a streamlined workflow to optimize operational efficiency is equally important. Fig. 1 presents a concrete example of such a workflow, illustrating the step-by-step process from image acquisition, through virtual surgical planning, to final surgical application. This workflow emphasizes clear responsibilities, critical check points, and seamless collaboration.

## 6 Case examples

In the following case examples, we demonstrate the clinical implementation of 3D technology in the reconstruction of bone defects of the upper extremity.

### 6.1 Thumb reconstruction

**Fig. 2:**
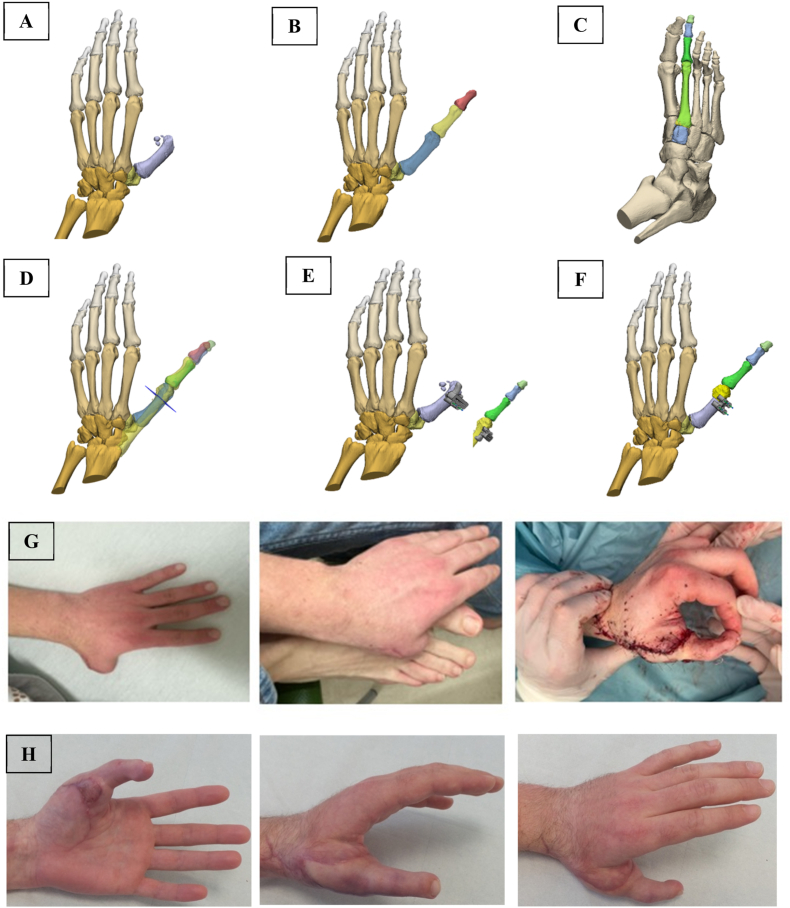
**Thumb reconstruction** **A** 20-year-old male patient underwent toe-to-thumb reconstruction due to an amputation of his right thumb through the first metacarpophalangeal (MC1) joint. (A) Three-dimensional (3D) reconstruction of the affected hand. (B) The thumb of the unaffected hand virtually straightened and aligned over the affected hand. (C) 3D reconstruction of the contralateral foot with the second toe virtually straightened. (D) The second toe, including metatarsal 2 bone (MT2), virtually straightened and superimposed over the virtually reconstructed thumb; saw planes for both MC1 and MT2 were determined. (E) Patient-specific saw guides designed for osteotomy of MC1 and MT2. (F) Patient-specific repositioning guide. (G) Pre- and post-reconstruction photographs. (H) Clinical photographs at 3-months postoperative follow-up.

**Fig. 3:**
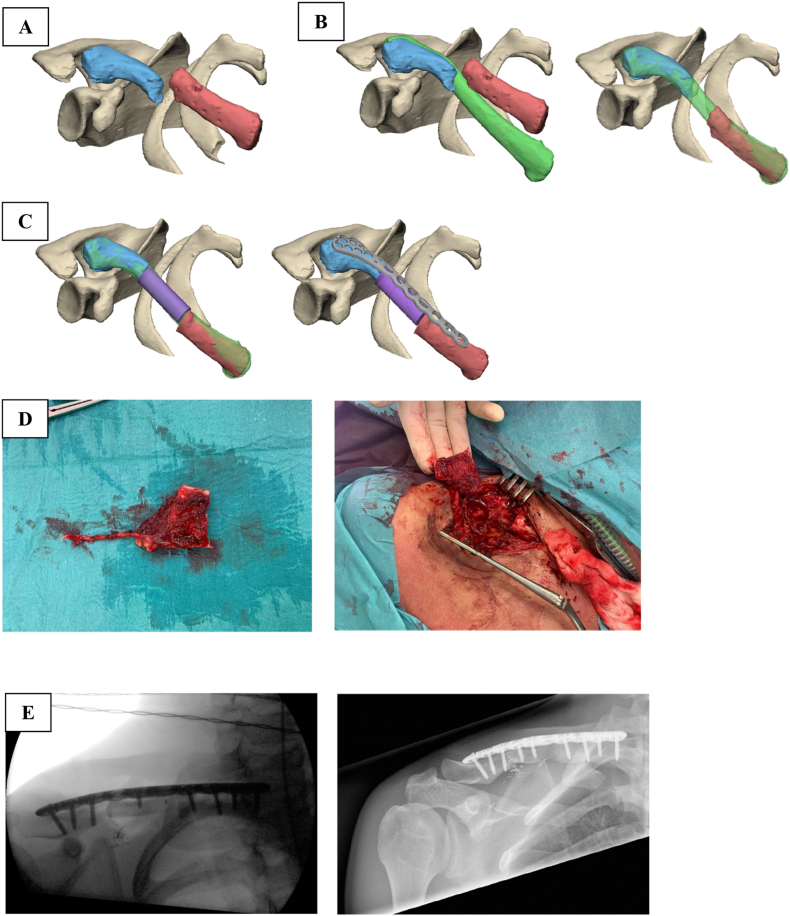
**Clavicula reconstruction** A 20-year-old female patient underwent vascularized bone reconstruction due to persistent malunion of the right clavicle. (A) Three-dimensional (3D) reconstruction of the affected clavicula. (B) Contralateral clavicula superimposed over the affected side and virtually repositioned. (C) Osteotomy planes, scapular tip graft and osteosynthesis plate position virtually determined. (D) intraoperative photographs of the scapular tip graft before (left) and after (right) reconstruction. (E) Radiographs before (left) and 6 months after reconstruction (right).

A 20-year-old furniture builder with an amputation of his thumb through the metacarpophalangeal joint. Using 3D virtual planning, the second toe was transferred to reconstruct the thumb. The steps were as follows: 1) CT scans from the affected hand, unaffected hand and the contralateral foot were made, 2) The thumb of the unaffected hand was virtually straightened and superimposed over the affected hand for the ideal length and angles (Fig. 2), 3) The foot's second toe was virtually straightened and superimposed over the thumb to determine the saw planes for both the metacarpal 1 bone (MC1) as well as the metatarsal 2 bone (MT2), and 4) Patient-specific saw guides and repositioning guides were designed.

After dissecting the flap, the proximal part of MT2 and the tip of MC1 were removed using the saw guides and placed onto each other using the reposition guide. Osteosynthesis was achieved with a plate and the result was a precise fit in one cut of the bone, saving significant time.

### 6.2 Clavicula reconstruction

A 20-year-old vocational sports student presented with a persistent malunion of the right clavicula following multiple previous surgeries. After the last procedure, in which compromised bone segments were removed and internal fixation was placed, the patient developed a complete brachial plexus palsy. The osteosythesis plate was subsequently removed, after which the neurological function fully recovered. Initial CT scans of the affected right clavicle and the contralateral healthy clavicle revealed a 5 cm bone defect (Fig. 3). Reconstruction was planned using a scapula tip graft, harvested following the technique previously described by Gomez-Martinez de Lecea et al.^9^ Subsequently, a CT scan of the scapula (donor side) was performed and a 3D model was generated to precisely determine the osteotomy positions for both the donor and recipient sites. Radiographic evaluation at six months postoperatively confirmed complete bone consolidation. The patient successfully resumed sports training without any functional deficits.

## 7 Future perspectives

Multidisciplinary collaboration is important for centers to properly implement technical innovations, such as 3D technology, into surgical practice. Three-dimensional technology in upper extremity surgery offers opportunities to improve surgical treatment. However, suboptimal use of the technology can lead to inefficient care and technological complexities. Technical physicians play a crucial role in multidisciplinary collaborations due to their integrative medical and technical competencies and should therefore be standard members of the team.^10^ Their expertise in segmentation, modeling, and workflow management is indispensable for ensuring the safe, efficient, and effective implementation of 3D technology in surgical practice.

Another critical step is to overcome the current limitations of data visualization. Although 3D technology enables surgical virtual planning in 3D, the output is traditionally displayed on two-dimensional screens. This not only limits the surgeons' ability to fully appreciate complex anatomy but also hinders patients’ understanding when their condition is presented in a flat, two-dimensional format. Extended Reality (XR) technology could bridge this gap in the future. This technology encompasses Virtual Reality (completely virtual environment), Augmented Reality (real environment with digitally overlayed images) and Mixed Reality (interaction and manipulation of both the virtual and real environment). In the pre-operative setting, XR could serve as a tool for both surgical training and patient education, offering realistic simulations and improving informed consent discussions. Intra-operatively, XR may enable navigation by overlaying surgical plans directly on the patient, while also integrating soft tissue visualization.

Lastly, the incorporation of artificial intelligence (AI) can enhance data visualization and intraoperative guidance. AI enables automated segmentation, producing high-quality anatomical reconstructions almost instantaneously, allowing their use even in emergency settings where time is critical. Intra-operatively, advanced AI capabilities will further enrich the XR experience by providing real-time visualization of bone resection areas, continuous updates on the extent of resection, and immediate alerts when surgical instruments approach critical (neurovascular) structures. Such capabilities could fundamentally transform intraoperative decision-making, moving toward a intelligent, real-time surgical environment.

## 8 Conclusion

The implementation of 3D technology in upper extremity surgery enhances both preoperative planning and intraoperative procedures. It has the potential to improve surgical outcomes, reduce the need for secondary surgeries, shorten operative time, and minimize radiation exposure. This technology has broad applicability, ranging from routine procedures to complex reconstructions. A well-coordinated multidisciplinary team is essential to facilitate successful and accessible integration of 3D technology into clinical practice.

## Informed consent

Informed consent was obtained from the patients for their anonymized information to be published in this article.

## Grants and financial support

This research did not receive any specific grants from funding agencies in the public, commercial, or not-for-profit sectors.

## Declaration of competing interest

The authors declare that they have no known competing financial interests or personal relationships that could have appeared to influence the work reported in this paper.

## References

[1] Mankovich N.J., Cheeseman A.M., Stoker N.G. (1990). The display of three-dimensional anatomy with stereolithographic models. J Digit Imag.

[2] Mankovich N.J., Samson D., Pratt W. (1994). Surgical planning using three-dimensional imaging and computer modeling. Otolaryngol Clin North Am.

[3] Mendonça C.J.A., Guimarães RM. da R., Pontim C.E. (2023). An overview of 3D anatomical model printing in orthopedic trauma surgery. J Multidiscip Healthc.

[4] Caiti G., Dobbe J.G.G., Strackee S.D. (2020). Computer-assisted techniques in corrective distal radius osteotomy procedures. IEEE Rev Biomed Eng.

[5] Kong L., Yang G., Yu J. (2020). Surgical treatment of intra-articular distal radius fractures with the assistance of three-dimensional printing technique. Medicine.

[6] Chen C., Cai L., Zheng W. (2019). The efficacy of using 3D printing models in the treatment of fractures: a randomised clinical trial. BMC Muscoskelet Disord.

[7] Meng M., Wang J., Sun T. (2022). Clinical applications and prospects of 3D printing guide templates in orthopaedics. J Orthop Translat.

[8] van Doremalen R.F.M., van der Linde R.A., Kootstra J.J. (2021). Can 3D-printing avoid discomfort-related implant removal in midshaft clavicle fractures? A four-year follow-up. Arch Orthop Trauma Surg.

[9] Gomez-Martinez de Lecea C., Schweizer R., Thor A., Rodriguez-Lorenzo A. (2022). Five-step Scapula tip flap harvesting for oromaxillofacial defects reconstruction. Plast Reconstr Surg.

[10] Groenier M., Spijkerboer K., Venix L. (2023). Evaluation of the impact of technical physicians on improving individual patient care with technology. BMC Med Educ.

